# Primary adenocarcinoma of the renal pelvis: A case report

**DOI:** 10.1097/MD.0000000000042655

**Published:** 2025-05-30

**Authors:** Pei Qin, Chunben Xu, Yuetao Zhao

**Affiliations:** a Department of Oncology, No. 923 Hospital of PLA, Nanning, Guangxi Zhuang Autonomous Region, China.

**Keywords:** adenocarcinoma of the renal pelvis, case report, chemotherapy, immunotherapy, surgery

## Abstract

**Rationale:**

Primary adenocarcinoma of the renal pelvis is a rare malignant tumor, with approximately 100 cases reported in the English literature. This malignancy is characterized by high-grade aggressiveness, diagnostic challenges at initial presentation, and advanced disease at diagnosis, leading to poor overall prognosis. Historically, treatment has focused on surgical resection combined with chemotherapy, but documented cases remain scarce. The emergence of immune checkpoint inhibitors (ICIs) necessitates the exploration of novel therapeutic combinations to improve survival outcomes. We present a case of primary renal pelvic adenocarcinoma managed with a multimodal approach integrating radical surgery, adjuvant chemotherapy, and concurrent ICI therapy, resulting in prolonged survival.

**Patient concerns:**

A 61-year-old male presented with a 3-month history of recurrent left flank and abdominal pain. He reported no hematuria or weight loss but expressed concern about worsening pain unresponsive to prior interventions. A left nephrostomy performed at an outside hospital revealed a renal pelvic mass, prompting a referral for further evaluation.

**Diagnoses:**

Previous left nephrostomy and drainage at a local hospital revealed a renal pelvic mass, with a biopsy confirming adenocarcinoma. Contrast-enhanced computed tomography demonstrated abnormal thickening of the left renal pelvis, calyces, and upper-middle ureter, retroperitoneal lymphadenopathy, postnephrostomy changes, and bilateral renal calculi. Serum carbohydrate antigen 19-9 (CA19-9) was elevated to 431.793 U/mL.

**Interventions:**

Laparoscopic radical left nephroureterectomy with partial cystectomy was performed on January 12, 2023, revealing a thickened left ureter and retroperitoneal lymphadenopathy. Pathology confirmed an 8 cm moderately differentiated adenocarcinoma infiltrating renal/ureteral/bladder tissues. Immunohistochemistry: CK(+)/CK7(+)/CEA(+), Ki-67 20% to 30%. Intraoperative pirarubicin instillation was administered.

**Outcomes:**

Postoperative stage IV disease received 2 cycles of adjuvant docetaxel. Disease progression (April 2023) prompted gemcitabine + tislelizumab (6 cycles), achieving a partial response (lymph node regression; CA19-9: 431.8→22.4 U/mL). Acute kidney injury (August 2023) necessitated tislelizumab monotherapy (3 cycles). Lymph node recurrence (January 2024) led to gemcitabine-tislelizumab rechallenge (2 cycles), followed by tislelizumab-pemetrexed (3 cycles) for stable disease. Bilateral lung metastases emerged post-treatment discontinuation (January 2025), treated with toripalimab-lenvatinib (1 cycle). The final follow-up (March 2025) documented 28-month survival (Eastern Cooperative Oncology Group 1).

**Lessons:**

This case underscores the imperative for multimodal integration (surgery, chemotherapy, and ICIs) to optimize survival in advanced renal pelvic adenocarcinoma while highlighting the necessity of dynamic therapeutic adaptation—including regimen rechallenge and tyrosine kinase inhibitor combinations—to address recurrence and resistance. Proactive toxicity management (e.g., dose de-escalation for renal injury) and rigorous biomarker-driven surveillance (serial CA19-9 tracking with 3-month imaging) emerge as critical strategies to balance efficacy and safety in this aggressive malignancy.

## 1. Introduction

Transitional cell carcinoma and squamous cell carcinoma are the adenocarcinoma of the renal pelvis most common types of epithelial malignancies of the renal pelvis, accounting for 85% to 90% and 10% to 15%, respectively, while adenocarcinoma of the renal pelvis occupies <1%.^[1]^ Due to no typical clinical symptoms and imaging features, preoperative diagnosis of adenocarcinoma of the renal pelvis is difficult. Its pathogenesis remains unclear, and it is generally believed that local long-term inflammatory stimuli, such as calculus and infection, are etiological factors.^[2]^ Radical surgery is the first choice for the treatment of adenocarcinoma of the renal pelvis with a poor prognosis, and chemoradiotherapy and immunotherapy can ameliorate the prognosis. After undergoing radical surgery, chemotherapy, and immunotherapy, the patient with adenocarcinoma of the renal pelvis reported herein was in stable condition and still under maintenance therapy.

## 2. Case presentation

### 2.1. Chief complaints

A 61-year-old male with repeated swelling pain in the left waist and abdomen for more than 3 months.

### 2.2. History of present illness

An elderly male patient experienced swelling pain in the waist and abdomen more than 3 months ago. In November 2022, the patient was diagnosed with “bilateral renal calculi and left hydronephrosis” in the local hospital, and underwent “left nephrostomy and drainage.” A left renal pelvis mass was found intraoperatively, and moderately differentiated adenocarcinoma was confirmed by pathological biopsy. The patient visited our hospital for surgery.

### 2.3. History of past illness

The patient had a history of bilateral renal calculi for more than 20 years, and denied a history of diabetes, heart disease, hypertension, surgery, and trauma.

### 2.4. Personal and family history

The patient denied exposure to chemical agents and radiation, and had an occasional history of smoking and no history of drinking. He denied a family history of hereditary diseases and infectious diseases, and a family history of similar diseases.

### 2.5. Physical examination

The patient denied exposure to chemical agents and radiation, and had an occasional history of smoking and no history of drinking. He denied a family history of hereditary diseases and infectious diseases, and a family history of similar diseases.

### 2.6. Laboratory examinations

#### 2.6.1. Carbohydrate antigen 19-9

431.793 U/mL, CA50 quantitation: > 180 U/mL, cancer antigen 125 (CA-125): 95.545 U/mL, and creatinine: 130 μmol/L.

### 2.7. Imaging examinations

**Figure 1.**
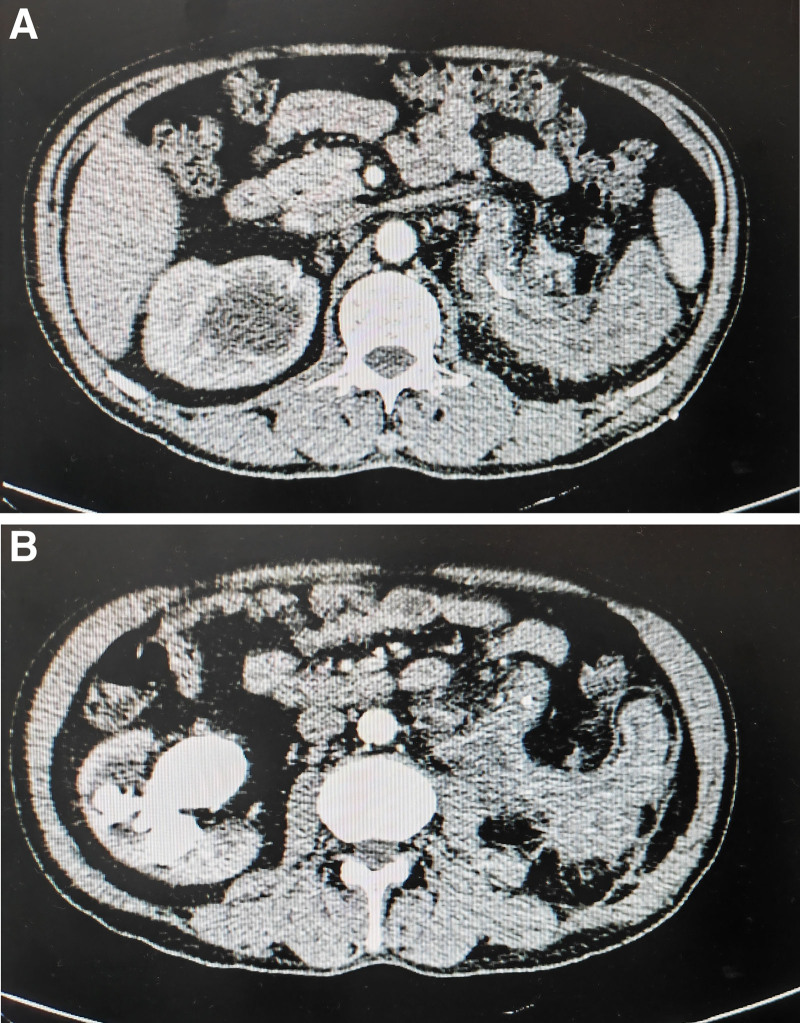
Computed tomography examination. (A) Patchy soft tissue density shadow in the left kidney region with heterogeneous enhancement, and patchy low density shadow without enhancement inside; (B) multiple lymph node enlargement around the lesion and beside the abdominal aorta, with a larger short diameter of about 1.1 cm, and heterogeneous thickening of the upper-middle segment of left ureter with obvious enhancement.

The computed tomography (CT) examination of the urinary system on January 4, 2023 showed abnormal morphology of the left kidney, unclear structures of the renal pelvis and calyx, patchy soft tissue density shadow in the left kidney region with heterogeneous enhancement, patchy low density shadow without enhancement inside, multiple lymph node enlargement around the lesion and beside the abdominal aorta, with a larger short diameter of about 1.1 cm, and heterogeneous thickening of the upper-middle segment of left ureter with obvious enhancement (Fig. 1). These findings were considered abnormal changes in the left kidney, renal pelvis and calyx, and upper-middle segment of ureter, malignancy complicated with retroperitoneal lymph node metastasis, changes after left nephrostomy, and multiple calculi in both kidneys. Thoracic and abdominal CT showed no obvious abnormality.

## 3. Final diagnosis

Carcinoma of the left renal pelvis with retroperitoneal lymph node metastasis. Bilateral renal calculi.

## 4. Treatment

On January 12, 2023, laparoscopic radical resection of carcinoma of the left renal pelvis + ureterolysis + cystoscopy + partial cystectomy was performed. During surgery, obvious thickening and hardening of the upper-middle segment of left ureter were found, and the left kidney and retroperitoneal hilar lymph nodes and the ureter were resected. Postoperative pathology: Moderately differentiated adenocarcinoma of the renal pelvis, the maximum diameter of cancer tissue = 8 cm, infiltration of surrounding renal tissues, involvement of the whole ureter and part of bladder tissues, intravascular tumor thrombus (−), and nerve invasion (−). Immunohistochemistry: CK (+), CK7 (+), CK20 (+), CEA (+), CK5/6 (partially +), P53 (−), and Ki-67 (20%–30% +). Intravesical instillation of pirarubicin hydrochloride for injection was performed once intraoperatively.

## 5. Outcome and follow-up

The patient was diagnosed with stage IV adenocarcinoma of the renal pelvis following left nephrectomy, complicated by preexisting right nephrolithiasis and renal insufficiency. Postoperative management comprised 2 cycles of adjuvant docetaxel monotherapy (75 mg/m² on day 1, q21 days). Surveillance CT in April 2023 demonstrated disease progression marked by enlargement and increased numbers of retroperitoneal and left iliac paravascular lymph nodes. This prompted therapeutic escalation to gemcitabine (1000 mg/m² on days 1 and 8, q21 days) combined with tislelizumab immunotherapy (200 mg per dose, q21 days) over 6 cycles, achieving partial radiographic response with reduction in mediastinal/retroperitoneal lymphadenopathy and significant CA19-9 decline. However, acute kidney injury following right renal calculus extraction in August 2023 necessitated transition to tislelizumab monotherapy for 3 cycles. Disease recurrence manifested in January 2024 via progressive retroperitoneal/iliac lymphadenopathy, leading to gemcitabine-tislelizumab rechallenge for 2 cycles. Continued progression observed in April 2024 warranted regimen modification to tislelizumab (200 mg q21 days) plus pemetrexed (500 mg/m² day 1, q21 days) for 3 cycles, achieving stable disease. Treatment nonadherence preceded the development of bilateral pulmonary metastases detected in January 2025. Salvage therapy with toripalimab (240 mg q21 days) and lenvatinib (8 mg daily) was initiated for 1 cycle before subsequent treatment discontinuation. Telephone follow-up in March 2025 documented persistent cough without additional symptoms, with an overall survival duration of 28 months since initial diagnosis.

## 6. Discussion

**Figure 2.**
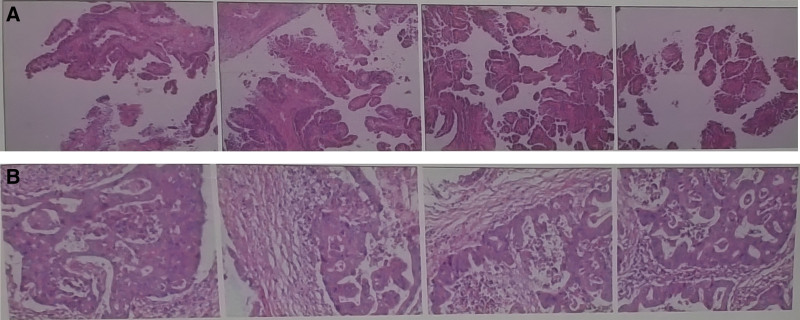
Moderately differentiated adenocarcinoma of the renal pelvis. (A) HE: 100×; (B) HE: 200×.

Renal pelvis tumors account for 3.1% to 7.7% of renal tumors, and they mostly originate from epithelium, most of which are urothelial carcinoma, and adenocarcinoma is rare.^[3]^ Clinically, most retroperitoneal adenocarcinomas originate from gastrointestinal tissues, so preoperative and intraoperative comprehensive examinations are required to exclude primary abdominal tumors in the diagnosis of primary adenocarcinoma of the renal pelvis. CT scan of the patient reported herein after admission showed space-occupying lesions in the left kidney region and no space-occupying shadow at other sites, and postoperative pathology suggested moderately differentiated adenocarcinoma of the renal pelvis, so the patient was definitely diagnosed with primary adenocarcinoma of the renal pelvis (Fig. 2).

The pathogenesis of adenocarcinoma of the renal pelvis remains unclear, which is possibly related to chronic stimuli such as urinary calculi, hydronephrosis, inflammation and leukoplakia.^[4]^ Due to long-term renal pelvic calculi and pyelitis, transitional cells develop pyelitis glandularis or pyelitis cystica, with nested acinar structure and secretion of mucus, and smooth muscle hyperplasia occurs around the acinus. Pyelitis glandularis and pyelitis cystica belong to chronic proliferative inflammation, which can evolve into adenocarcinoma.^[5]^ The patient reported herein had a history of renal calculi for more than 20 years, which was also a possible cause of this disease.

Adenocarcinoma of the renal pelvis has no specific early symptoms, and gross hematuria is the most common and initial symptom. Dull pain in the waist affects about one-third of patients, and other rare symptoms include fever, weight loss, anemia and urinary tract irritation, which are similar to the clinical symptoms of renal calculi, so it tends to be easily misdiagnosed as renal calculi.^[6]^ The patient reported herein had a history of renal calculi. He visited the local hospital due to waist pain. The ultrasound examination in the local hospital suggested left renal calculi with hydronephrosis. During “left nephrostomy and drainage,” mass of the left renal pelvis was found, and moderately differentiated adenocarcinoma of the renal pelvis was confirmed by biopsy. It suggests that we should be alert to the possibility of urinary system tumor in patients with long-term repeated lumbago or hematuria in clinical practice. CT and MRI are of great significance for preoperative diagnosis, which can better display the size, site and shape of tumors, and preliminarily judge tumor invasion status and pelvic lymph node enlargement. Urinary cytology and fluorescence in situ hybridization are often applied for preoperative screening and postoperative follow-up, and ureteroscopy and endoscopic pathological biopsy are helpful for diagnosis.^[7]^ The significance of tumor markers for the diagnosis of primary adenocarcinoma of the renal pelvis has not been clarified. It has been pointed out in the literature that carcinoembryonic antigen, CA19-9 and CA-125 significantly increase in adenocarcinoma of the renal pelvis.^[8]^ CA19-9 in the patient reported herein significantly increased and rapidly decreased after surgery and chemotherapy, similar to the report results by Kato et al^[9]^ that the levels of serum CEA and CA19-9 decrease simultaneously after tumor resection.

Radical nephroureterectomy with bladder cuff excision constitutes the standard therapeutic approach for renal pelvic adenocarcinoma, with strict adherence to tumor containment protocols during surgery being imperative to prevent intraoperative rupture and subsequent metastatic dissemination.^[10]^ The clinical utility of neoadjuvant chemotherapy and postoperative adjuvant chemoradiation remains controversial. Current evidence supports consideration of adjuvant radiotherapy for margin-positive or margin-involved cases to mitigate locoregional recurrence risk (Level 2B recommendation), though conflicting reports exist demonstrating recurrence within 12 months postradiation, suggesting limited therapeutic efficacy in this malignancy.^[11]^ Standardized chemotherapy regimens remain undefined, with most protocols adapted from urothelial carcinoma guidelines. The gemcitabine-cisplatin/carboplatin combination represents a commonly employed first-line option demonstrating potential progression-free survival (PFS) benefits.^[12]^ Retrospective analyses by Wang et al^[13]^ documented divergent outcomes in 2 primary upper urinary tract adenocarcinoma cases: 1 developing retroperitoneal/hepatic metastases at 28 months without adjuvant chemotherapy versus another maintaining 19-month DFS with adjuvant treatment. Liu et al further reported 6-month metastasis-free status using adjuvant nab-paclitaxel in a moderately differentiated renal pelvic adenocarcinoma.^[14]^ Emerging immunotherapeutic strategies show variable promise, with tislelizumab achieving median overall survival (OS) of 9.8 months and objective response rate (ORR) of 24.8% in second-line advanced urothelial carcinoma.^[15]^ The phase III CheckMate 274 trial demonstrated nivolumab’s superiority over placebo in adjuvant settings for high-risk urothelial cancers (including renal pelvic subtypes), with significant disease-free survival (DFS) improvement (21 vs 10.9 months; HR 0.70) and manageable toxicity (17.9% grade 3–4 events), particularly in PD-L1-positive subgroups.^[16]^ HER2-targeted therapies hold potential given 58.4% HER2 overexpression rates, with trastuzumab showing activity in amplification-positive tumors^[17]^ and antibody-drug conjugates demonstrating efficacy in advanced disease.^[18]^ Combination strategies such as camrelizumab (200 mg q3w) plus famitinib (20 mg daily) exhibit encouraging outcomes (ORR 30.6%, DCR 63.9%, median OS 12.9 months) in multicenter trials.^[19]^ The present case highlights sequential therapeutic challenges: initial adjuvant docetaxel monotherapy (2 cycles) followed by gemcitabine-tislelizumab combination (6 cycles) achieving partial response, subsequent renal impairment necessitating tislelizumab monotherapy (3 cycles) with disease progression, rechallenge failure with gemcitabine-tislelizumab (2 cycles), pemetrexed-tislelizumab stabilization (3 cycles), treatment discontinuation culminating in pulmonary metastases, and eventual salvage therapy with toripalimab-lenvatinib (ongoing at March 2025 follow-up).

## 7. Conclusion

This case highlights critical insights into the management of renal pelvic adenocarcinoma – a rare malignancy with an estimated incidence of 0.5% to 1.2% among renal tumors, frequently complicated by concurrent nephrolithiasis (present in 60% to 80% of cases) that contributes to diagnostic delays through stone misidentification. Multimodal preoperative evaluation incorporating advanced cross-sectional imaging (contrast-enhanced CT/MRI) and urinary cytology is essential for differential diagnosis. Curative-intent radical surgery remains the therapeutic cornerstone, while adjuvant systemic therapies guided by molecular biomarkers – including HER2 overexpression, mismatch repair (MMR) status, NTRK gene fusions, and tumor mutational burden – demonstrate potential to prolong PFS and optimize oncologic outcomes. Emerging evidence supports the integration of next-generation sequencing into postoperative pathological workflows to enable precision medicine strategies. Prospective multicenter trials validating targeted therapies (e.g., HER2-directed ADCs) and immunotherapy combinations are strongly recommended, with parallel emphasis on patient-reported outcomes and quality-of-life metrics to balance survival benefits against treatment-related toxicities in this clinically challenging population.

## Author contributions

**Conceptualization:** Yuetao Zhao.

**Data curation:** Pei Qin.

**Formal analysis:** Pei Qin.

**Investigation:** Chunben Xu, Yuetao Zhao.

**Methodology:** Pei Qin, Chunben Xu.

**Resources:** Pei Qin.

**Software:** Chunben Xu.

**Supervision:** Yuetao Zhao.

**Validation:** Pei Qin.

**Writing – original draft:** Pei Qin.

**Writing – review & editing:** Yuetao Zhao.
